# From pure testicular Teratoma to primitive neuroectodermal tumor: Successful management of Teratoma malignant transformation with widespread metastases in a young adult

**DOI:** 10.1016/j.eucr.2024.102830

**Published:** 2024-08-22

**Authors:** Navid Masoumi, Ali Amani-Beni, Elnaz Ataei, Atoosa Gharib, Amir Alinejad Khorram

**Affiliations:** a Department of Urology, Shahid Modarress Hospital, Shahid Beheshti University of Medical Sciences, Tehran, Iran; b Clinical Development Center of Shahid Modarres Hospital, Shahid Beheshti University of Medical Sciences, Tehran, Iran; c Clinical Research Development Center, Shahid Modarres Educational Hospital, Shahid Beheshti University of Medical Sciences, Tehran, Iran; d Department of Pathology, Shahid Modarres Hospital, Shahid Beheshti University of Medical Sciences, Tehran, Iran; e Urology Department, Shohada-e-Tajrish Hospital, Shahid Beheshti University of Medical Sciences, Tehran, Iran

## Abstract

Midline retroperitoneal masses in young males often present a diagnostic challenge, with metastases from testicular origins being a primary consideration. Beyond the initial pathology of testicular cancer, these masses can undergo transformation, including the development of teratomas. This report describes an unusual case of a calcified retroperitoneal mass originating from a testicular pure teratoma that underwent a rare transformation into a Primitive Neuroectodermal Tumor (PNET), comprising approximately 85 % of the tumor volume. The patient's successful treatment involved a comprehensive approach combining surgical resection and chemotherapy.

## 1 Introduction

Teratomas, which arise from the three germinal layers (mesoderm, endoderm, and ectoderm), have the potential to transform into a range of tumor types. These can include non-seminomatous germ cell tumors, sarcomas, squamous cell carcinomas, adenocarcinomas, carcinoid tumors, and primitive neuroectodermal tumors (PNETs).^1^^,^^2^.

Primitive Neuroectodermal Tumors (PNETs) are rare and aggressive neoplasms, notorious for their poor prognosis and resistance to cisplatin-based chemotherapy. Treatment of PNETs typically involves a comprehensive approach, encompassing surgical resection, radiotherapy, and chemotherapy, which are critical factors in determining a patient's outcome and prognosis. A notable and unusual occurrence is the transformation of teratoma into PNET, which can occur across ectodermal cell lines.^3^^,^^4^.

We present a case report of successful management of a retroperitoneal mass, which originated from a metastatic testicular pure teratoma which underwent a rare transformation into a primary neuroectodermal tumor (PNET), accounting for approximately 85 % of the tumor's volume.

## 2 Case report

A 36-year-old male presented to our urology clinic with a chief complaint of right flank pain and feeling of abdominal fullness. On physical examination, a mass-like structure was palpable on deep abdominal palpation. There was no tenderness and rest of the examination, including the neck, chest, and testes, was unremarkable.

The patient's laboratory results were significant for an elevated lactate dehydrogenase level at 795 U/L (normal range: 140–280 U/L), while alpha-fetoprotein and beta human chorionic gonadotropin levels were within normal ranges at 0.82 ng/mL and <2 mlU/mL, respectively.

Ultrasonographic evaluation of the testes revealed a heterogeneous lesion in the right testicle. The patient subsequently underwent a radical orchiectomy, and the pathology report confirmed a diagnosis of pure teratoma.

**Fig. 1:**
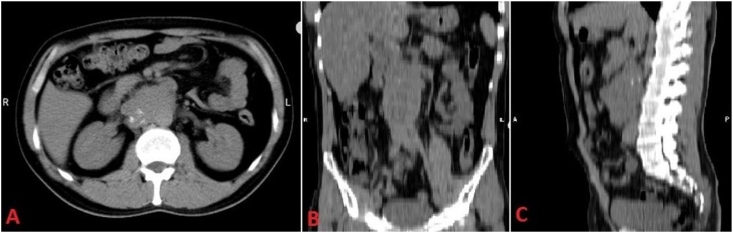
Retroperitoneal mass encasing the inferior vena cava and right renal vein with signs of calcification within the mass; Axial (A) Coronal (B) and Sagittal (C) sections of CT scan Imaging study.

A computed tomography (CT) scan revealed a retroperitoneal mass measuring 115 × 75 mm, encasing the inferior vena cava and right renal vein with signs of calcification (Fig. 1). Histological analysis of the mass showed benign cartilage tissue in biopsy.

**Fig. 2:**
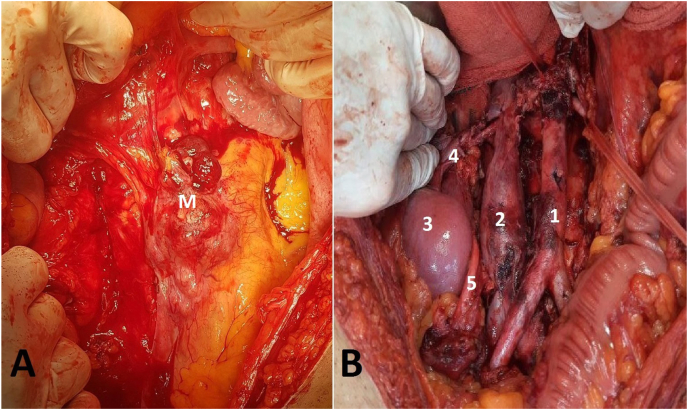
Pre- and post-operative imaging of the retroperitoneal area. Retroperitoneal mass before surgical resection; M: Mass (A). Retroperitoneal area after surgical resection of the retroperitoneal mass and lymph node dissection, with anatomical landmarks labeled: 1: Descending aorta, 2: Inferior vena cava, 3: Right kidney, 4: Right renal hilum, 5: Right ureter (B).

He was staged as IIB testicular cancer, and after collaboration in Uro-oncology multidisciplinary team, despite strong recommendations for primary chemotherapy in retroperitoneal involvement of testicular cancers, we prioritized surgical resection based on the primary pathology of the testicular mass, imaging findings of multiple calcifications, and biopsy results indicating cartilage tissue. Following the surgical resection, we await the final pathology report to determine the definitive diagnosis and guide our subsequent treatment plan. Fig. 2 shows the mass before resection and the retroperitoneal area after surgical resection of the mass and lymph node dissection.

**Fig. 3:**
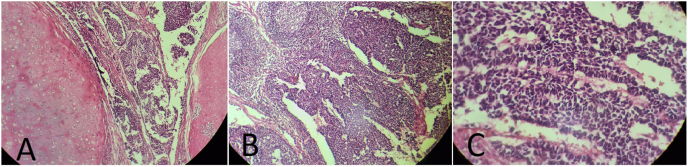
Microscopic examination of tumor. H&E × 40, Cartilaginous nodule and PNET (A), PNET metastatic to lymph node (B) and Rosette formation in PNET (C).

The permanent pathology report revealed that the tumor was a metastatic malignant germ cell tumor with a non-germ cell component. The non-germ cell component consisted of 85 % primitive neuroectodermal tumor (PNET) and 15 % pure cartilage teratoma (Fig. 3). A follow-up CT scan showed mediastinal/pulmonary and jugular masses, as well as multiple liver lesions, indicating metastatic disease.

The patient received a 17-cycle chemotherapy regimen, administered every three weeks, which alternated between two cycles. The regimen consisted of Regimen A (cyclophosphamide, vincristine, and doxorubicin) until the maximum dose of doxorubicin was reached, at which point it was replaced by dactinomycin; and Regimen B (isophosphamide and etoposide).

After completing 17 cycles of chemotherapy, the patient's treatment was deemed complete, and no residual metastatic masses were detected in follow-up CT scan. Given the rarity of the patient's case, we chose to use PET-CT scan as the most accurate diagnostic tool to assess the response to treatment. The scan revealed no evidence of primary or metastatic tumors, indicating a successful outcome from the chemotherapy regimen. Currently, the patient remains disease-free after three months of follow-up, but longer-term follow-up is needed to accurately assess his disease-free survival and overall survival.

## 3 Discussion

Teratomas are a unique type of tumor that arises from the germinal layers and can comprise various tissues, including bone, cartilage, teeth, hair, or muscle. In approximately half of all non-seminomatous germ cell tumors (NSGCTs), teratomas are present. Notably, in most patients with metastatic mature teratoma, evidence of teratoma is typically evident in the primary tumor. Furthermore, there is a well-established risk of teratoma transformation into other tumor types.^1^^,^^2^.

The report of cartilage tissue in the biopsy of the retroperitoneal mass of the patient presented in this article suggested the possible origin of the teratoma. Despite normal testicular examination, a teratoma tumor was eventually detected in the right testis. our treatment team opted for a multidisciplinary approach, prioritizing surgical resection of the retroperitoneal mass and retroperitoneal lymph node dissection before systemic treatment. This led to the diagnosis of PNET transformation in the retroperitoneal metastatic tumor.

The fifth edition of the World Health Organization's (WHO) Classification of Urinary and Male Genital Tumors introduced a change in terminology, renaming "primitive neuroectodermal tumor" as "embryonic-type neuroectodermal tumor".^1^ PNETs are rare, aggressive, and malignant. peripheral PNETs can manifest in various locations, such as the paravertebral area in the retroperitoneum, chest wall, pelvis, and extremities. Due to their highly aggressive nature, a multidisciplinary approach to management is strongly recommended, typically involving wide resection and combination therapies such as chemoradiotherapy.^3^.

Despite the traditional belief that PNET is resistant to cisplatin-based chemotherapy, recent studies have demonstrated that chemotherapy regimens commonly used for Ewing's sarcoma treatment are effective in managing PNETs that arise from transformed teratoma^4^. Despite the resistance of PNET to cisplatin-based chemotherapy^4^^,^^5^, a chemotherapy regimen including cyclophosphamide, vincristine and doxorubicin alternating with isophosphamide and etoposide has been used effectively in the management of PNET. This treatment regimen is considered PNET-specific chemotherapy regimen and PNET is highly chemo-sensitive to it^5^^,^^6^. In the patient presented in this article, we also used this treatment regimen, which showed a positive response.

A recent case report describes a similar case that was treated with a comprehensive treatment approach, including radical orchiectomy, retroperitoneal lymph node dissection, and adjuvant chemotherapy. The patient received a combination of vincristine, adriamycin, and cyclophosphamide (VAC) alternating with ifosfamide and etoposide (IE) therapy.^7^ The successful management of the patient presented in our article also involved a multimodal treatment strategy, encompassing surgical resection and chemotherapy.

## 4 Conclusion

PNET is a rare transformation of teratoma, and recent studies have reported positive outcomes for chemotherapy in PNETs arising from transformed teratomas. We present a case of a patient with testicular metastatic teratoma that underwent PNET transformation in a retroperitoneal mass. This patient was successfully managed with a multimodal treatment strategy involving surgical resection and chemotherapy. Our case highlights the importance of considering the testicular origin of unusual retroperitoneal masses.

## Ethics

Patient informed consent was obtained to publish his information. The patient's private information remained confidential with the researchers.

## Financial support and sponsorship

None.

## CRediT authorship contribution statement

**Navid Masoumi:** Writing – review & editing, Validation, Supervision, Methodology. **Ali Amani-Beni:** Writing – original draft, Investigation. **Elnaz Ataei:** Writing – original draft, Visualization, Conceptualization. **Atoosa Gharib:** Writing – original draft, Validation. **Amir Alinejad Khorram:** Writing – review & editing, Methodology, Conceptualization.

## Declaration of competing interest

The authors report no conflicts of interest in this work.
